# Mortality Risks in Patients With Schizophrenia in Shanghai: A Longitudinal Cohort Study

**DOI:** 10.62641/aep.v54i3.2022

**Published:** 2026-06-15

**Authors:** Yu Zhou, PanPan Zhang, Feng Wang, WeiBo Zhang, YiHua Jiang

**Affiliations:** ^1^Disease Prevention and Control Department, Minhang District Mental Health Center, 201112 Shanghai, China; ^2^Changqiao Subdistrict Community Health Service Center, 200231 Shanghai, China; ^3^Shanghai Mental Health Center, Shanghai Jiao Tong University School of Medicine, 200030 Shanghai, China

**Keywords:** schizophrenia, mortality, cohort studies, epidemiologic studies

## Abstract

**Background:**

Schizophrenia is a severe mental disorder associated with significantly higher mortality risk than the general population, constituting a major public health challenge. Clarifying temporal trends in mortality among patients with schizophrenia helps inform targeted clinical and public health interventions. To assess changes in mortality rates over the last 2 years in patients with schizophrenia from one district of Shanghai, China.

**Methods:**

This longitudinal cohort study assessed the mortality of individuals with schizophrenia (n = 5234) from April 1, 2021, to March 31, 2023. The early cohort (up to March 31, 2022) contained 5207 individuals and the late cohort (up to March 31, 2023) contained 5114 individuals. Partitions of Pearson’s chi-square statistic were used to conduct pairwise comparisons. A multivariable Cox proportional hazards model was used to assess the effects of different factors on mortality risk. Data were drawn from the official Shanghai database used for following-up individuals with severe mental health disorders.

**Results:**

The total number of deaths was 370. Of these, 120 occurred in the early cohort (2.30% of the cohort) and 250 in the late cohort (4.89% of the cohort). Individuals with schizophrenia in the late cohort had a 5.08-fold increased mortality risk than individuals in the general population. In the early cohort, mortality rates remained relatively stable (0.29%–0.48%). There were peaks in mortality rates (2.23%) in December 2022 and January 2023. Men had a higher risk of mortality than women (hazard ratio [HR], 1.294; 95% confidence interval [CI], 1.035–1.618; *p* = 0.024). Age was a contributing factor to the mortality of people with schizophrenia (HR, 1.048; 95% CI, 1.040–1.056;* p* < 0.001). Being unmarried (HR, 1.956; 95% CI, 1.522–2.513;* p* < 0.001), divorced (HR, 1.481; 95% CI, 1.010–2.170; *p* = 0.044), or widowed (HR, 1.334; 95% CI, 0.958–1.856; *p* = 0.088) was associated with a high risk of mortality. Age of onset (HR, 1.046; 95% CI, 1.037–1.054;* p* < 0.001) and illness duration (HR, 1.044; 95% CI, 1.037–1.051;* p* < 0.001) were associated with high mortality risk. The late cohort showed a higher mortality rate than the early cohort (HR, 2.427; 95% CI, 1.467–4.014; *p* = 0.001). The mortality of patients with schizophrenia was not significantly related to education level, economic status, or symptom onset.

**Conclusions:**

These findings highlight trends in schizophrenia mortality since April 2021. Compared with the general population, individuals with schizophrenia had higher mortality risk, particularly adults aged ≥65 years and women with low education.

## Introduction

According to the China Statistical Yearbook 2011 [1], by the end of 2010, the total population of China was 1.341 billion. A total of 7.16 million people in China had been affected with schizophrenia during their lifetime [2]. The prevalence of schizophrenia in China in 2010 was 0.5%. The China Mental Health Survey was set up in 2012 [3], and 32,552 respondents had completed the survey by 2015. The survey showed that the weighted lifetime prevalence of schizophrenia and the weighted 12-month prevalence of schizophrenia were both 0.6% (95% confidence interval [CI] 0.1–1.0) [3].

Clearly, people with schizophrenia have a mortality risk that is two to three times that of the general population [4]. Over the last 3 years, most research on the mortality of individuals with schizophrenia has been conducted in Israel [5], the UK [6], and the USA [7], with a lack of research on this topic from China.

A severe mental health disorder database system (the SHMD system) was developed in 1999 in Shanghai. The Shanghai Municipal Mental Health Regulations were passed in 2001 and became effective from April 7, 2002 [8]. The Standing Committee of the Shanghai Municipal People’s Congress passes information about patients (including name, address, and suspected symptoms) with severe mental health disorders to the mental health centers in their respective administrative districts. After diagnosis by district mental health center physicians, a patient record is generated by physicians or nurses at the respective community health centers. The records are used for subsequent regular follow-ups according to patients’ conditions [9].

Patients in a stable condition are followed-up every 3 months. Patients in a mostly stable condition are followed up once a month. Those in an unstable condition are followed-up every 2 weeks. Fourteen criteria are used to assess patients’ conditions. Follow-up information includes patients’ symptoms, social functioning, medication information, treatment status, recovery method, and *in vitro* test results.

There are very few studies on mortality rates in individuals with schizophrenia in China. To the best of our knowledge, this is the first study to provide mortality rates for patients with schizophrenia in China after 2021. In this study, we aimed to assess whether the mortality rate of patients with schizophrenia in Shanghai has changed between 2021 and 2023. We further aimed to identify sociodemographic factors and disease status associated with mortality. Information about these factors may help to reduce deaths among people with schizophrenia.

## Materials and Methods

### Study Population

There are 18 hospitals that can provide mental disorder diagnoses in Shanghai. Since 2018, the Shanghai SHMD system has been integrated into the information systems of all 18 hospitals. Every new patient record of a schizophrenia diagnosis should be imported into the SHMD system, which generates reminders for the respective district mental health centers to start the follow-up process.

The study population was selected from the SHMD system. The inclusion criteria were (1) Patients registered before March 31, 2023; (2) A diagnosis of schizophrenia (F20) according to the International Statistical Classification of Diseases and Related Health Problems 10th Revision (ICD-10); and (3) Resident in a specific district of Shanghai. A total of 5555 patients met the inclusion criteria. The exclusion criterion was patients who died before March 31, 2021. In total, 321 patients died before March 31, 2021, and were excluded. Therefore, a total of 5234 schizophrenia patients were included in the study.

### Selection of Study Participants

We identified patients who were followed up from April 2021 to March 2022 (labelled the early cohort) and from April 2022 to March 2023 (labelled the late cohort).

### Database Field Definitions

Hospitalization Duration: The total number of consecutive days a patient is formally admitted to a medical institution (with a schizophrenia diagnosis, ICD-10 code F20) and receives inpatient treatment, calculated from the date of admission to the date of discharge (excluding day-case admissions or emergency department stays not leading to formal admission). Symptom Onset Timing: The interval between the first documentation of acute schizophrenia-related symptoms (e.g., hallucinations, delusions, disorganized speech, negative symptoms) by a qualified psychiatrist and the initiation of standardized treatment (inpatient or outpatient).

Grouping Criteria: Short hospitalization: ≤30 days (patients with mild-to-moderate symptoms responsive to acute treatment, or those discharged for community-based follow-up); Medium hospitalization: 31–90 days (patients with moderate symptoms requiring extended stabilization, or those with partial response to initial treatment); Long hospitalization: >90 days (patients with severe refractory symptoms, comorbid medical conditions requiring concurrent management, or lack of social support for community reintegration).

### Statistical Analysis

We analyzed the crude mortality ratios (CMR) for schizophrenia patients and individuals in the general population resident in the same district. Partitions of Pearson’s chi-square statistic were used to compare differences between schizophrenia patients and the general population between 2021 and 2023 using the following formula:

$$\alpha \,\text{′}=\frac{\alpha }{\frac{k\,\left(k-1\right)}{2}+1}=\frac{0.05}{\frac{4\,\left(4-1\right)}{2}+1},\alpha \,\text{′}=0.00714.$$

A multivariable Cox proportional hazards model was used to assess the effects of different factors on mortality risk. All other analyses were conducted using SPSS 23.0, IBM Corp (Armonk, NY, USA).

## Results

### Demographic Characteristics

A total of 5207 schizophrenia patients were included up to April 2021. Between April 2021 to March 2022, 120 patients died, and 5087 patients had left by the end of March 2022. Between April 2022 and March 2023, 27 patients were diagnosed with schizophrenia and added to the late cohort. A total of 5234 schizophrenia patients were included in the study; 5207 of these were in the early cohort (April 2021 to March 2022) and 5114 were in the late cohort (April 2022 to March 2023). The demographic characteristics are shown in Table 1A,1B. Most participants were older (≥65 years), had an educational level of middle school or below, and were not in poverty. Approximately 54% of male patients were unmarried and 36% were married. In contrast, 24% of female patients were unmarried and 59% were married. The total number of deaths was 370. Of these, 120 occurred in the early cohort (2.30% of the cohort) and 250 in the late cohort (4.89% of the cohort).

**Table 1A.** **Male baseline characteristics**.

Male		The early cohort (n = 2485)				The late cohort (n = 2438)			
		n	% Total	Deaths	% Group	n	% Total	Deaths	% Group
Age group	Below 15	0	0.00	0	NA	0	0.00	0	NA
	15–24	34	1.37	1	2.94	31	1.27	1	3.23
	25–34	173	6.96	0	0.00	143	5.87	0	0.00
	35–44	340	13.68	3	0.88	344	14.11	1	0.29
	45–54	386	15.53	5	1.30	377	15.46	7	1.86
	55–64	612	24.63	10	1.63	552	22.64	20	3.62
	65–74	686	27.61	19	2.77	713	29.25	60	8.42
	75–84	217	8.73	17	7.83	240	9.84	47	19.58
	85 above	37	1.49	5	13.51	38	1.56	14	36.84
Marital status	Unmarried	1360	54.73	38	2.79	1328	54.47	74	5.57
	Married	894	35.98	19	2.13	881	36.14	52	5.90
	Divorced	185	7.44	3	1.62	183	7.51	19	10.38
	Widowed	46	1.85	0	0.00	46	1.89	5	10.87
Education level	Illiteracy	53	2.13	5	9.43	48	1.97	5	10.42
	Primary school	293	11.79	10	3.41	285	11.69	27	9.47
	Middle school	1057	42.54	30	2.84	1028	42.17	72	7.00
	High school	628	25.27	11	1.75	619	25.39	28	4.52
	College and above	454	18.27	4	0.88	458	18.79	18	3.93
Economic status	Below the local poverty line	213	8.57	3	1.41	210	8.61	8	3.81
	Other	2272	91.43	57	2.51	2228	91.39	142	6.37

**Table 1B.** **Female baseline characteristics**.

Female		The early cohort (n = 2722)				The late cohort (n = 2676)			
		n	% Total	Deaths	% Group	n	% Total	Deaths	% Group
Age group	Below 15	5	0.18	0	0.00	4	0.15	0	0.00
	15–24	33	1.21	0	0.00	28	1.05	0	0.00
	25–34	140	5.14	0	0.00	126	4.71	1	0.79
	35–44	335	12.31	2	0.60	330	12.33	2	0.61
	45–54	480	17.63	3	0.63	454	16.97	5	1.10
	55–64	642	23.59	5	0.78	600	22.42	13	2.17
	65–74	699	25.68	12	1.72	724	27.06	32	4.42
	75–84	262	9.63	16	6.11	293	10.95	33	11.26
	85 above	126	4.63	22	17.46	117	4.37	34	29.06
Marital status	Unmarried	650	23.88	6	0.93	645	24.10	26	4.03
	Married	1607	59.04	30	1.88	1588	59.34	62	3.90
	Divorced	247	9.07	5	2.05	242	9.04	5	2.07
	Widowed	218	8.01	19	8.72	201	7.51	27	13.43
Education level	Illiteracy	179	6.58	13	7.26	170	6.35	24	14.12
	Primary school	388	14.25	12	3.09	378	14.13	28	7.41
	Middle school	1058	38.87	19	1.80	1044	39.01	50	4.79
	High school	612	22.48	10	1.63	602	22.50	14	2.33
	Collage and above	485	17.82	6	1.24	482	18.01	4	0.83
Economic	Below the local poverty line	186	6.83	4	2.15	183	6.84	2	1.09
	Other	2536	93.17	56	2.21	2493	93.16	118	4.73

### All-Cause Mortality

**Fig. 1.**
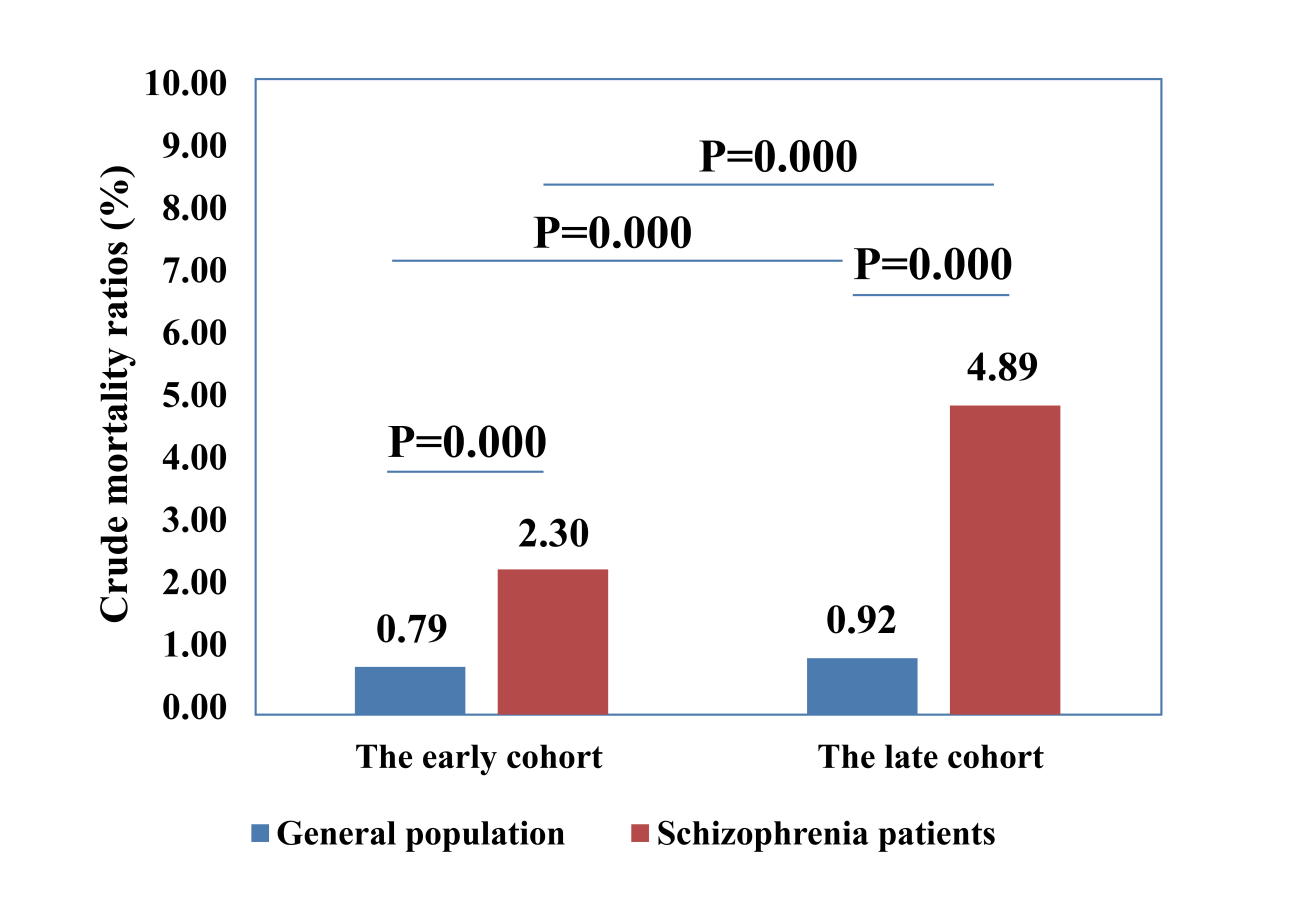
**Mortality among individuals with schizophrenia and the general population**.

Differences in CMR for different years for individuals with schizophrenia and for the general population are shown in Fig. 1. Individuals with schizophrenia in the early cohort had a mortality risk that was 2-fold that of the general population. From 2022, the CMR of both individuals with schizophrenia and the general population increased; there was a statistically significant difference between any of the two groups (*p* < 0.001). However, in the late cohort, individuals with schizophrenia had a 5.08-fold increased mortality risk compared with the general population.

### Trends in Schizophrenia Mortality From April 2021 to March 2023

**Fig. 2.**
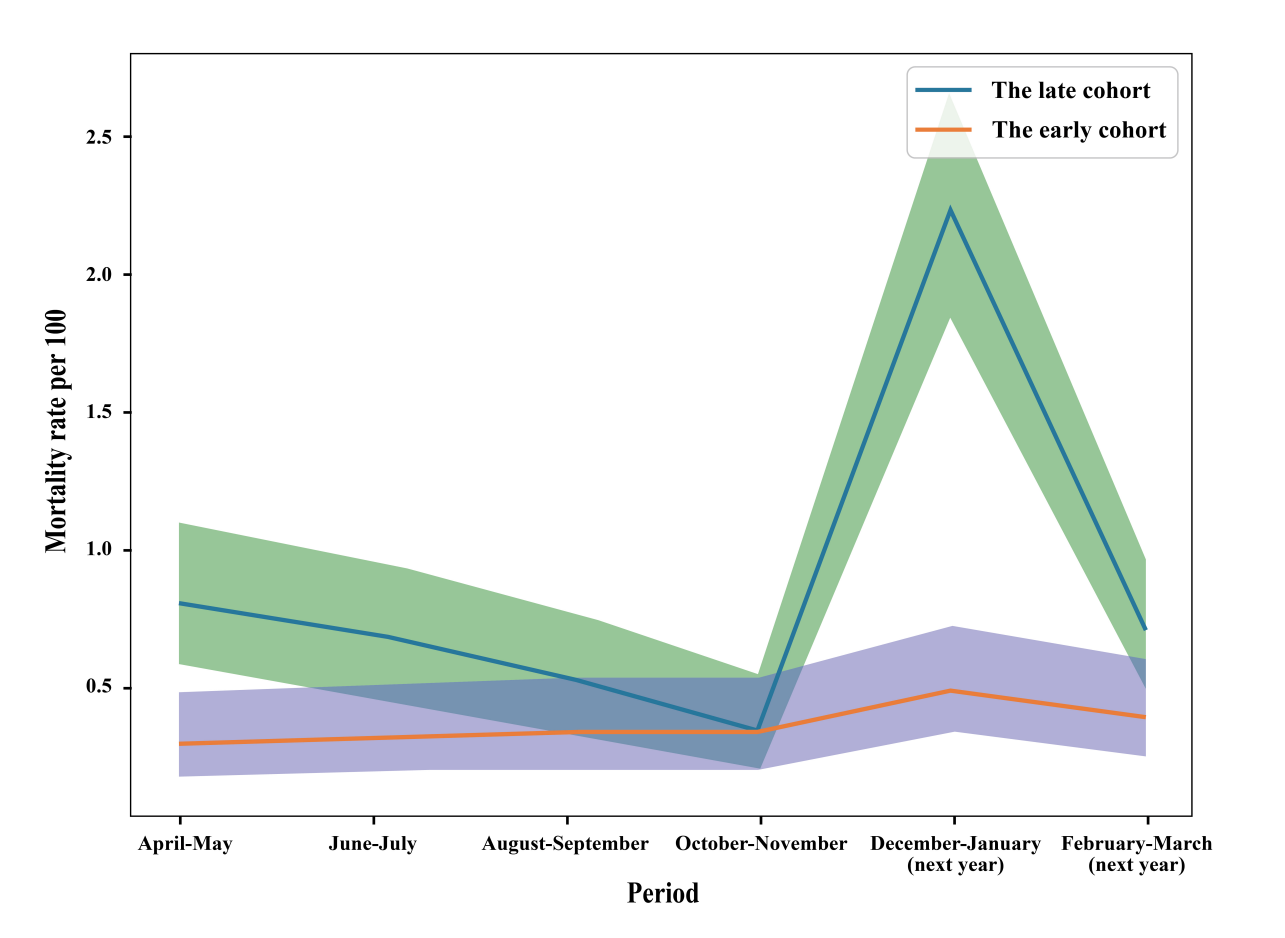
**Mortality trends over 2 years (2022–2023) for individuals with schizophrenia**.

Changes in mortality patterns for individuals with schizophrenia in the early and late cohorts were assessed (Fig. 2). Overall, mortality rates for the late cohort were higher than those for the early cohort for the whole period; the respective ranges were 0.33%–2.23% vs 0.29%–0.48%. In the early cohort, mortality rates remained relatively stable. There were peaks (2.23%) in mortality rates in December 2022 and January 2023. Mortality then dropped to 0.70% in February 2023 and March 2023.

### Factors Underlying the Association Between Schizophrenia and Mortality

Men with schizophrenia showed a higher risk of mortality than women with schizophrenia (hazard ratio [HR] 1.294; 95% CI, 1.035–1.618; *p = *0.024). Age was a contributing factor to mortality risk (HR, 1.048; 95% CI, 1.040–1.056; *p* < 0.001). Being unmarried (HR, 1.956; 95% CI, 1.522–2.513; *p* < 0.001), divorced (HR, 1.481; 95% CI, 1.010–2.170; *p* = 0.044), or widowed (HR, 1.334; 95% CI, 0.958–1.856; *p* = 0.088) was associated with a high risk of mortality. Age of onset (HR, 1.046; 95% CI, 1.037–1.054; *p* < 0.001) and duration of illness (HR, 1.044; 95% CI, 1.037–1.051; *p* < 0.001) were associated with high risk of mortality. The late cohort had a higher risk of mortality than the early cohort (HR, 2.427; 95% CI, 1.467–4.014; *p* = 0.001). The mortality of patients with schizophrenia was not significantly related to education level, economic status, or symptom onset (see Table 2 for details).

**Table 2.** **Different characteristics associated with mortality in patients with schizophrenia (HRs and 95% CIs)**.

Characteristics		β	SE	Wald	df	*p*	HR	95% CI for HR
Sex								
	Female							
	Male	0.258	0.114	5.131	1	0.024	1.294	1.035–1.618
Age		0.046	0.004	142.817	1	<0.001	1.048	1.040–1.056
Marital status								
	Married							
	Unmarried	0.671	0.128	27.483	1	<0.001	1.956	1.522–2.513
	Divorced	0.392	0.195	4.045	1	0.044	1.481	1.010–2.170
	Widowed	0.288	0.169	2.918	1	0.088	1.334	0.958–1.856
Education level								
	Collage and above							
	Illiteracy	NA	NA	1.832	1	0.176	NA	NA
	Primary school	NA	NA	0.316	1	0.574	NA	NA
	Middle school	NA	NA	0.272	1	0.602	NA	NA
	High school	NA	NA	0.000	1	0.988	NA	NA
Economic								
	Other							
	Below the local poverty line	NA	NA	2.176	1	0.140	NA	NA
Symptom onset								
	14 days (acute)							
	15–30 days	NA	NA	0.509	1	0.476	NA	NA
	(subacute)							
	31 days (chronic)	NA	NA	0.031	1	0.859	NA	NA
Age of onset		0.045	0.004	114.269	1	<0.001	1.046	1.037–1.054
Duration of illness		0.043	0.003	153.672	1	<0.001	1.044	1.037–1.051
Group								
	Early cohort							
	Late cohort	0.887	0.257	11.924	1	0.001	2.427	1.467–4.014

CI, Confidence Interval; DF, Degrees of Freedom; HR, Hazard Ratio; NA, Not Applicable values in SPSS; SE, Standard Error.

## Discussion

Several previous studies indicate that schizophrenia patients are at increased risk of mortality compared with the general population [10, 11]. A study in Manitoba, Canada, showed that the CMR for individuals with schizophrenia was double that of those without (20.00% vs 9.37%) after 10 years follow-up [12]. A nationwide study in Finland found that the all-cause CMR of persons with schizophrenia remained stable (2.3%–2.9% per year) during 1984–2014 [13]. An 8-year longitudinal study in Shandong province of China showed that the CMR was 2155.51 per 100,000 years from 2012 to 2019 [9]. The present findings showed that the CMR in the early cohort was similar to that of previous studies.

We also calculated the Standardized Mortality Ratio (SMRs) of Shanghai’s general population. The general population data were obtained from the Shanghai Municipal Bureau of Statistics (Years 2022 and 2023) [14, 15], which provides age- and gender-stratified mortality rates. The SMR results show that in 2022 there was an SMR of 2.93, and 3.00 in 2023. The present study demonstrated that individuals with schizophrenia in the late cohort had a 5.08-fold increase in mortality risk compared with the general population. Consistent with the results of an Israeli study (OR, 3.27, 95% CI, 1.39–7.68; *p* < 0.001), these findings indicate that individuals with schizophrenia were three times more likely to die than controls [16]. Similar findings have been reported in studies from other countries, such as Denmark [17] and South Korea [18]. Our study showed that male patients had a higher mortality risk than female patients; in the late cohort, the gap in case fatality rates between men and women was even greater. One systematic review reported that male sex was a likely contributor to increased mortality risk in people with severe mental illness [19]. In both cohorts, mortality in male and female patients correlated with age. A rapid growth in mortality rates was found for individuals aged ≥65 years, and there was a greater increase in mortality with age in the late cohort compared with the early cohort. Similar findings for older people in the general population were described in a United States National Center for Health Statistics data brief [20]. For male patients in the late cohort, we found that there was no effect on mortality regardless of education level. In the same cohort, the results indicated a significantly increased mortality risk in women with the lowest level of education; illiterate patients had approximately a 3-fold increased risk of death than those with a college degree. No previous study has shown that illiterate female schizophrenia patients have a higher mortality risk than other individuals with schizophrenia after 2020. A Swedish study found that a subgroup in the national cohort with higher educational attainment had fewer adverse outcomes, including long hospitalization and death [21]. Being unmarried, divorced, or widowed was associated with a high risk of mortality of schizophrenia in the present study. A 14-year follow-up study in rural southwest China found that marriage can be instrumental in improving family-based support and caregiving, thereby facilitating community living for persons with schizophrenia [22].

Schizophrenia requires lifelong treatment [23], even when symptoms have subsided. People with schizophrenia in Shanghai have full access to medical treatment as part of their basic human rights. Hospital inpatient care is one of the best choices for patients with severe symptoms or those whose families cannot take care of them at home. We suggest that one possible explanation of the higher mortality risk in individuals with schizophrenia is social isolation [24]. However, it is important to note that the SHMD system does not include information about severity of schizophrenia or comorbid medical conditions, which may be additional risk factors for mortality [25]. It can be difficult to obtain reliable mortality data. Because of the lack of data on causes of death in our study, analyzing only all-cause mortality may mask the risk differences in specific causes of death.

Taken together, the present findings comprehensively demonstrate that individuals with schizophrenia have a greater mortality risk than the general population, and this difference was greater in individuals in the late cohort. Thus, better strategies and more support should be provided for individuals with severe mental disorders.

## Conclusions

This study highlights mortality trends since April 2021. We found that patients with schizophrenia, particularly those aged ≥65 years and women with low education, were at a higher risk than the general population.

## Availability of Data and Materials

The data supporting this study’s findings were derived from the Shanghai Mental Health Monitoring and Data (SHMD) system. Given the data contains sensitive personal health information of patients with schizophrenia, raw data will not be made publicly available in accordance with the Personal Information Protection Law of the People’s Republic of China and relevant ethical regulations to safeguard patient privacy. Qualified researchers seeking access to confidential data may submit a written application, including a detailed research proposal, data protection compliance statement, and proof of ethical approval (if applicable), to the Ethics Committee of Minhang District Mental Health Center (No. 2500 Zhahang Road, Minhang District, Shanghai; Tel: +86-54840696), which will review requests to balance patient rights and legitimate academic research. Non-identifiable supporting materials, excluding raw patient data, are available from the corresponding author upon reasonable request.
